# Smartphone addiction and creativity in Chinese undergraduates: a moderated mediation model analysis

**DOI:** 10.3389/fpsyt.2025.1570547

**Published:** 2025-06-13

**Authors:** Li Cheng, Han Xie

**Affiliations:** ^1^ Hubei Preschool Education Research Center, Hubei University of Education, Wu Han, China; ^2^ Teacher Education Research Center, Hubei University of Education, Wu Han, China

**Keywords:** undergraduates, smartphone addiction, creativity, depression, rumination

## Abstract

**Objectives:**

In the digital era, the relationship between smartphone addiction and creativity among Chinese undergraduates has drawn increasing attention. This study aimed to explore how depression mediates the relationship between smartphone addiction and creativity, and how positive rumination moderates this mediating effect, with the goal of clarifying the underlying psychological mechanisms and providing insights for promoting creativity and mental well - being among this population.​

**Methods:**

A cross - sectional study was carried out. Undergraduate students from three Chinese provinces were sampled through a questionnaire distributed via the Wenjuanxing online platform. The questionnaire measured smartphone addiction, depression, creativity, and positive rumination. A total of 401 valid responses were obtained. Moderated mediation analysis was employed to examine the relationships among these variables.​

**Results:**

The analysis showed that smartphone addiction significantly predicted depression, but had no significant direct effect on creativity. Depression negatively predicted creativity. It was confirmed that depression mediated the relationship between smartphone addiction and creativity. Moreover, positive rumination moderated the relationship between depression and creativity, and a protective effect was observed when the level of positive rumination was higher. The moderated mediation model proposed in this study was validated.

**Conclusions:**

The study successfully validated the moderated mediation model, indicating that positive rumination weakens the negative impact of depression on creativity in the context of smartphone addiction. The findings suggest that positive rumination can potentially help alleviate the adverse effects of excessive smartphone use on the creative thinking of Chinese undergraduates.

## 1 Introduction

In the information age, creativity has become one of the most valuable assets for contemporary undergraduates (1). Creativity is critical to academic achievement, driving innovation, solving problems, and adapting to rapidly changing social demands (2–5). In an era of knowledge explosion and technological innovation, where college students are expected to develop creativity to become future leaders and change agents, smartphones offer a prime opportunity (6). Undergraduates can use smartphones as learning, communication, and creative expression tools, leveraging apps and features to explore ideas, connect widely, and potentially create their own digital content, thereby unlocking their potential and contributing to digital evolution (7, 8). However, the proliferation and excessive use of smartphones may negatively affect this critical ability. Research indicates that smartphone overuse can reduce the volume of several brain regions associated with cognition, impulse control, emotions and behaviors, and brain reward systems, which may adversely affect an individual’s memory and learning capabilities (9). Therefore, the relationship between smartphone addiction and creativity needs to be explored among Chinese undergraduates, and the underlying mechanisms must be identified. This study aims to provide a theoretical foundation for developing strategies to mitigate the potential negative effects of smartphone addiction on creativity and foster a more balanced and healthy use of technology among Chinese undergraduates.

Smartphones provide numerous forms of gratification, such as sociability, entertainment, information retrieval, time management, and social identity maintenance (10–13). Smartphones have become an integral part of daily life, and research has shown that certain people become so attached to their devices that they experience separation anxiety when without them (14, 15). Previous studies have linked smartphone addiction and mental health issues, particularly depression (16, 17). Depression, which is characterized by persistent feelings of sadness and lack of interest or pleasure in activities, has been identified as a critical factor impeding creative expression (18). This study contributes to the literature by providing a more nuanced understanding of the mechanisms linking technology use, mental health, and cognitive function. By dissecting the relationships between smartphone addiction, depression, and creativity, we offer insights into the psychological consequences of technology use and potential avenues for intervention.

Smartphones, primarily relying on internet-based apps, are rendered versatile and ubiquitously carried by their portability and app-installation capacity. Therefore, the portability of smartphones, the variety and appeal of apps, instant gratification, social interaction, multitasking, and personalized recommendations all work together to make it easier for users to develop mobile phone addiction. Lin et al. (19) regarded smartphone addiction as a form of technological addiction. Smartphone addiction symptoms may differ from those of substance addiction. Smartphone addiction has similarities to DSM-5 substance-related disorders in terms of compulsive behavior, functional impairment, withdrawal, and tolerance (20). Meta-analysis results derived from 24 countries show that smartphone addiction is increasing across the world (21). Smartphone addiction has received extensive attention, especially among undergraduates (22–25).

According to the Cognitive Resource Theory (26), individuals have finite cognitive resources that must be allocated among different cognitive tasks. In the context of smartphone addiction, individuals addicted to smartphones tend to engage in activities such as excessive social media use and compulsive gaming, which consume a substantial portion of their cognitive resources (27, 28). Consequently, when confronted with creative tasks that demand focused attention, information integration, and the generation of novel ideas, the paucity of available cognitive resources, resulting from the preoccupation with smartphones, impedes the creative process and the manifestation of creativity (29, 30). For instance, during creative writing endeavors or design undertakings, interruptions and distractions caused by smartphone addiction can disrupt the continuity and profundity of cognitive processing, thereby thwarting the full exploitation of creative capabilities.

Recent psychological research has provided empirical support for the significant association between smartphone use and creativity. Olson et al. (31) surveyed 48,000 participants and found that a negative correlation was seen in a small sample, larger samples showed only weak correlations. Guan et al. (32) studied 998 college students and found a positive relationship between mobile phone use and creative ideation, mediated by critical thinking and creative self-efficacy; however, this study did not focus on addiction behavior. Li et al. (33) provided neuroimaging evidence that individuals with smartphone addiction have reduced brain activity and weaker functional connectivity during creative idea generation, suggesting that smartphone addiction adversely affects creativity. Other researches had demonstrated that smartphone addiction negatively impacts brain regions associated with cognitive control, a process central to creative idea generation (34, 35). Overall, the latest evidence supports the negative effects of smartphone addiction on creativity.

Depression is a psychological disorder manifesting as a major cause of negative emotion (36). Meta-analytic evidence demonstrates that depression, as a behavioral outcome, is consistently and positively associated with smartphone addiction across diverse geographical regions and subject populations (37). And a common consequence of depression is a significant decline in productivity (38). Moreover, negative or neutral mood states are less effective than positive affect in fostering creative performance (39). Cognitive neuroscience has found that the medial prefrontal cortex, which is essential for associative processing and creativity (40), displays abnormal activity in individuals with depression (41). This abnormality can lead to overinhibition of the medial temporal lobe, thereby constraining the activation of crucial associative networks for creativity (42). Furthermore, cognitive flexibility, which is markedly higher in highly creative individuals, tends to be reduced in individuals with depression compared with controls (43). The negative correlation between problem-solving ability and psychopathology further complicates the assumed link between depression and reduced creative performance (44, 45). In summary, depression may be negatively correlated with creativity among college students, and it plays a mediating role between smartphone addiction and creativity.

Although creativity may be linked to smartphone addiction through depression, not all people with depression experience decreased creativity (46). This heterogeneity in outcomes could be explained by theoretical frameworks emphasizing the dual nature of cognitive processes in depression,such as perseverative cognition theory. Perseverative cognition theory holds that the results of perseverative cognition are uncertain and depend on the value of the thinking content and one’s mood during reflection (47, 48). Perseverative cognition is characterized by repetitive or chronic activation in response to one or more psychological stressors, representing a common stress response pattern (47). Rumination, in particular, constitutes a form of perseverative cognition (49). In previous studies, rumination has been defined as repetitive thinking about negative events and regarded as a negative cognitive process (50, 51). However, some researchers, such as Martin and Tesser (52) believe it can be positive. Frone (53) explicitly proposed the concepts of negative and positive work rumination. Moreover, Yang et al. (54) developed the Positive and Negative Rumination Scale, and found that scores on the positive rumination subscales were positively and significantly associated with life satisfaction and optimism. Inspired by the above-mentioned studies, exploring the buffering effects of positive rumination has great value.

In the process of researching rumination, some researchers have already proposed the concept of positive rumination and developed the corresponding scale (55), defining it as the tendency of individuals to generate a positive emotional state due to repeatedly pondering over their own positive qualities, positive emotional experiences, and a good living environment. Rumination can have beneficial effects in multiple respects. From a cognitive processing perspective, it is an in-depth cognitive exploration of past experiences and unachieved goals. Through this repetitive thinking, individuals may dissect the details of their actions and decisions, similar to how scientists meticulously analyze experimental processes and results during research impasses, which makes understanding the self-regulatory functions of positive rumination important (53). This could potentially lead to discovering overlooked aspects and novel insights, thereby opening new avenues for problem-solving and progress. With respect to self-growth and learning, rumination is a crucial mechanism of self-reflection (56). When individuals ruminate on past mistakes or unfinished tasks, they can extract valuable lessons and identify areas for improvement. For instance, students can ruminate on their learning strategies and exam performance to pinpoint knowledge gaps and subsequently modify their study methods, ultimately contributing to their personal and academic development.

From the perspective of emotion processing, emotion regulation (ER) theory (57) illustrates how individuals manage emotional intensity, duration, and expression through cognitive/behavioral strategies to adapt to environmental demands. Existing research has identified positive rumination as a critical emotion regulation mechanism: it enhances, sustains, and amplifies positive emotions by actively reflecting on positive experiences (58–61). This process strengthens neural representations of positive affect via cognitive restructuring, forming a cycle of adaptive emotion and cognition. As a strategy that amplifies positive affect through cognitive restructuring, positive rumination may counteract depression’s tendency to deplete cognitive resources, thereby preserving the mental flexibility essential for creative idea generation. This theoretical link motivates our hypothesis: positive rumination moderates the relationship between depression and creativity.

This study investigated the mediating role of depression and the moderating role of rumination between smartphone addiction and creativity among undergraduate students. Based on the literature review, three hypotheses were formulated for this study:

*H1: Smartphone addiction is negatively related to creativity.*
*H2: Depression mediates the relationship between smartphone addiction and creativity. Additionally, smartphone addiction is positively related to depression, which, in turn, is negatively related to creativity.*
*H3: Positive rumination moderates the relationship between depression and creativity.*

**Figure 1:**
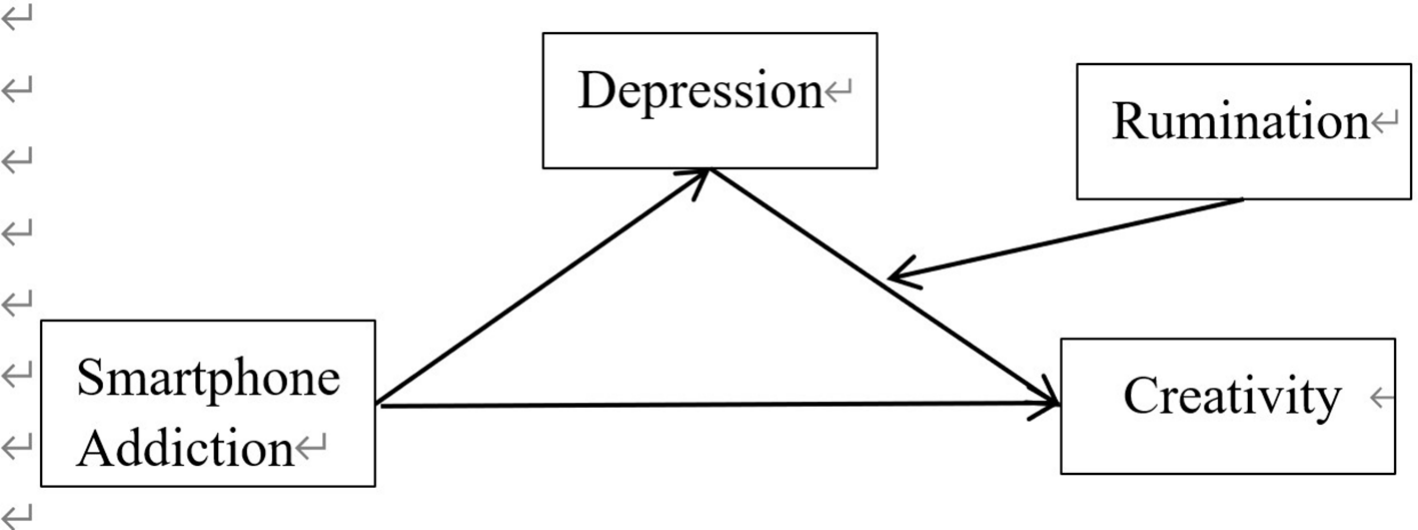
The conceptual model.

Based on the above theoretical considerations and hypothetical deductions, this study proposes the conceptual model shown in **Figure 1**.

## 2 Method

### 2.1 Participants and procedures

A convenience sampling method was employed in the current study. To reduce potential selection bias and enhance sample diversity, several targeted strategies were implemented during the recruitment process. First, three universities in Hubei Province were purposively selected to represent varying educational tiers: (a) “Double First-Class” institutions, (b) provincial key universities, and (c) local colleges, thereby ensuring diversity in both academic resources and student backgrounds. Second, within each selected class, students were stratified based on gender and academic performance (categorized into top, middle, and bottom tertiles according to GPA). Class supervisors then randomly invited participants from each stratum, ensuring proportional representation across subgroups. These measures were taken to minimize the limitations inherent in convenience sampling and to enhance the reliability and generalizability of the study findings.These measures were taken to reduce the selection bias caused by the convenience sampling method and ensure the reliability and applicability of the study results. The data were collected via the Wenjuanxing platform between August 5 and September 5, 2024. Participants took approximately 15 minutes to complete the questionnaire. A total of 451 responses were initially obtained.After excluding participants with regular response patterns and abnormal response times, 401 (64 male, 337 female) valid questionnaires were retained for analysis, yielding an effective response rate was 87.75%. **Table 1** shows the demographic characteristics of the participants.

**Table 1:** Demographic characteristics of the participants (n = 401).

Variables	N	%
Gender		
Male	64	16.0
Female	337	84.0
Grade		
Freshman	106	26.4
Sophomore	129	32.2
Junior	152	37.9
Senior	14	3.5
Academic Performance		
Excellent	84	20.9
Good	237	59.1
Average	60	15.0
Poor	20	5.0

### 2.2 Measures

#### 2.2.1 Demographic characteristics

This study’s demographic characteristics included gender, grades, only-child status, and hometown. Gender was set as dummy variables (male = 1, female = 0). Grade level (including freshman, sophomore, junior, and senior) was captured with a single-choice question. Only child status (yes or no) and hometown (urban or rural area) were incorporated to comprehensively understand the participants’ background characteristics relevant to the research. Previous findings indicate that the demographic variables mentioned above may be linked to the current main variables (62, 63). Thus, demographic variables were controlled as covariables in the data analysis process.

#### 2.2.2 Smartphone addiction

Based on Leung’s (64) Mobile Phone Addiction Index, the questionnaire contains 17 items, ranging from “completely disagree” scored as 1 point to “completely agree” scored as 5 points. The higher the score, the more evident the addiction tendency. Cronbach’s α in this study was 0.91.

#### 2.2.3 Depression

Depression was assessed using the Depression Anxiety and Stress Scale-21 developed by Lovibond et al. (65). The depression subscale has seven items scored on a 4-point Likert scale from 0 (“not applicable”) to 3 (“always applicable”). Higher scores indicate more severe depression. Cronbach’s α in this study was 0.87.

#### 2.2.4 Positive rumination

The rumination was assessed by the Ruminative Response Scale developed by Nolen-Hoeksema et al. (66). This scale includes three dimensions–symptom-related rumination, reflective pondering, and brooding–with a total of 22 items. It adopts a 4-point Likert scoring method, ranging from 1 (“never”) to 4 (“always”). A higher total score indicates more severe ruminative thinking. In this study, we focused on the role of positive rumination; through reflective pondering, one may focus on analyzing and exploring issues from multiple angles and levels in a rational and critical way of thinking, and distinguishing various viewpoints and possibilities during the thinking process. Therefore, we analyzed the reflective pondering dimension of this scale. Cronbach’s α in this study was 0.88.

#### 2.2.5 Creativity

Creativity was assessed using the Individual Innovation Scale developed by Scott and Bruce (67). The scale has six items scored on a 5-point Likert scale ranging from 1 (“not applicable”) to 5 (“always applicable”). One of the example items is “I am able to come up with creative ideas.” For all 6 items, Cronbach’s α in this study was 0.94.

### 2.3 Data analyses

Common method bias testing was employed to assess the quality and reliability of the research data and eliminate artificial covariance between variables that may arise from using the same method or source to collect data, thereby ensuring the accuracy and objectivity of the research findings. Subsequently, Pearson’s correlation analysis was employed to explore the correlation between the study variables. A mediation and moderated mediation model was then implemented using the PROCESS macro (Model 14) for SPSS version 3.4. We first examined the mediating role of depression in the association between smartphone addiction with creativity and then examined the moderating effect of rumination on the mediating role of depression in the link between smartphone addiction and creativity. Mediation and moderated mediation models were interpreted using standardized path estimates (β) and squared-multiple correlations (R^2^). All analyses were conducted using SPSS version 24.0.

## 3 Results

### 3.1 Common method bias

The data collection method of this study was to issue questionnaires completed by Chinese Undergraduates according to their actual conditions. Considering this study used self-report measures, there were concerns about common method bias (68). Harman’s single-factor method was used for the calculations and tests. The test results showed that the maximum factor variance interpretation rate was 34.06%, which did not reach the critical standard of 40%, indicating no serious common method bias.

### 3.2 Descriptive statistics and correlation analysis

As shown in **Table 2**, smartphone addiction was positively correlated with depression (*r* = .304, *p* <.01) and rumination (*r* = .183, *p* <.01). Depression was negatively correlated with creativity (*r* = -.190, *p* <.01) and positively correlated with rumination (*r* = .617, *p* <.01).

**Table 2:** Correlations among the variables.

Variables	M	SD	1	2	3	4
1. Smartphone Addiction	2.74	0.72	1			
2. Depression	1.63	0.57	.304^**^	1		
3. Creativity	3.26	0.52	.010	-.190^**^	1	
4. Positive Rumination	1.94	0.68	.183^**^	.617^**^	-.055	1

SA, Smartphone Addiction; DE, Depression; CR, Creativity, PR, Positive Rumination.

**p<.01 (two-tailed).

### 3.3 Testing the mediating role of depression

As shown in **Table 3**, the predictive effect of smartphone addiction on depression was significant (*β* = 0.239, SE = .038, *p* <.001). The direct effect of smartphone addiction on the dependent variable creativity was not significant (*β* = .053, SE = .037, *p* > 0.05), and the negative predictive effect of depression on creativity was significant (*β* = -.194, SE = .047, *p <*.001).

**Table 3:** Mediation and moderated mediation models (n = 401).

Process	Variables	Model 4			Model 14		
		β	SE	t	β	SE	t
1. Mediator variable Model (DE)	Constant	.974	.106	9.164^***^	.974	.106	9.164^***^
	SA	.239	.038	6.373^***^	.239	.038	6.372^***^
	R^2^ = .092, F (1,399) = 40.609				R^2^ = .092, F (1,399) = 40.609		
2.Dependent variable model (CR)	Constant	3.431	.101	31.223^***^	3.941	.238	16.593^***^
	SA	.053	.037	1.440	.053	.037	1.452
	DE	-.194	.047	-4.127^***^	-.623	.148	-4.222^***^
	PR				-.182	.106	-1.718
	DE*PR				.159	.058	2.738^*^
	R^2^ = .041, F (2,398) = 8.534				R^2^ = .065, F (4,396) = 6.909		

Process	Variables	Effect	BootSE	Boot95%CI
3. Conditional indirect effects	Low PR	-.101	.030	[-.164,-.047]
	High PR	-.050	.017	[-.083,-.018]

SA, Smartphone Addiction; DE, Depression; CR, Creativity; RU, Rumination. ***P<.001 (two-tailed).

### 3.4 Testing the moderated mediation model

**Figure 2:**
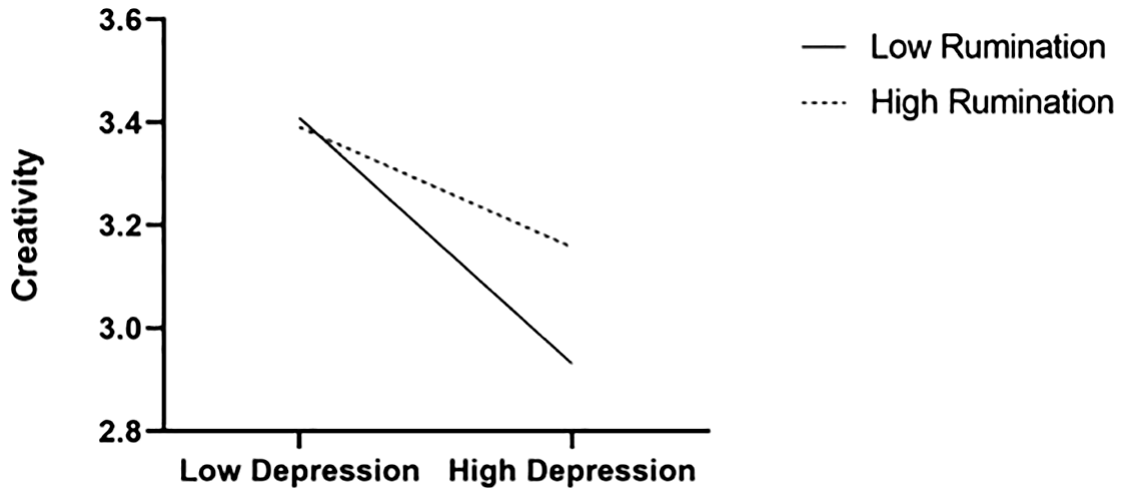
Moderated effect of rumination.

According to Model 14 (see **Table 3**), the interaction of depression and rumination was positively related to creativity, and the effect size was large (*β* = .159, SE = .058, p = .007). This result indicates that rumination moderates the relationship between depression and creativity. A simple slope analysis graph is shown in **Figure 2**.

## 4 Discussion

In the context of the exponential growth of digitalization in China (69), the present study was designed to comprehensively investigate the relationship between smartphone addiction and creativity in an undergraduate population. It further explored the mediating effect of depression, which was hypothesized to play a crucial role in bridging the association between smartphone addiction and creativity. Additionally, the moderating role of rumination was examined as it was postulated to influence the nature and strength of the relationship. The findings contribute to a deeper understanding of the underlying psychological and cognitive mechanisms governing the relationship between smartphone addiction and creativity. Such insights could pave the way for developing more effective preventive and therapeutic measures to address smartphone addiction and its associated consequences among college students, thereby promoting psychological well-being and academic success.

### 4.1 Smartphone addiction and creativity

There was no significant correlation between smartphone addiction and creativity, and the direct effect was not significant, which is inconsistent with Hypothesis 1. The reason for this result may be that the impact of smartphones on creativity hinges on usage patterns. Responsible use of information and communication technologies (ICTs) can boost creativity, yet addictive and dependent usage turns smartphones into tools that suppress creative expression. Instead of leveraging smartphones and the internet as aids for academic tasks while exercising creativity, many young people rely on them to complete assignments passively, bypassing creative thinking (70). This result may be more pronounced among individuals with pathological smartphone addiction.

### 4.2 The mediating role of depression

The results showed that depression positively correlated with smartphone addiction and negatively correlated with creativity. From theoretical and mechanistic perspectives, college students exhibit unique cognitive and psychological traits. Smartphone addiction in this group is characterized by excessive reliance on the information and entertainment provided by mobile devices, representing a misallocation of cognitive resources. This diverts attention and time from creativity-nurturing activities. For example, addicted students often interrupt academic and practical pursuits in favor of mobile stimuli such as social media and short videos. Such resource misallocation and reality evasion among college students can trigger depressive emotions (71, 72). Based on cognitive-behavioral theory, the negative outcomes of smartphone addiction, such as academic delay, social decline, and loss of self-efficacy, lead to negative self-perceptions and depressive feelings (73–76). Depression, which is a negative psychological state, significantly inhibits undergraduate creativity. Neuropsychologically, it correlates with prefrontal cortex dysfunction, which affects the cognitive functions essential for creativity (77). Depressed students face attention regulation imbalances, reduced motivation (as per motivation theory), and impaired cognitive flexibility, all of which impede their creative performance (78–80). In summary, depression mediates the relationship between mobile phone addiction and creativity among college students. These findings enrich our understanding of the underlying mechanisms, guiding research in this area toward greater refinement and systematization.

### 4.3 The moderating role of positive rumination

This study also found that positive rumination moderated the relationship between depression and creativity. Specifically, positive rumination can attenuate the negative impact of depression on creativity. This result enriches our understanding of the complex relationship between depression and creativity, and highlights the significance of individual cognitive coping styles.

From a cognitive appraisal theory perspective (81), positive rumination involves adaptive cognitive processing of internal and external stimuli. When individuals engage in positive rumination, they are more likely to constructively appraise their depressive experiences. Instead of passively succumbing to negative affect, they actively seek to reframe and make sense of it. This cognitive reappraisal process is hypothesized to activate neural pathways associated with positive affect and self-regulation, which, in turn, may counteract the neural circuitry underlying the negative impact of depression on creativity. In the context of depressive emotions, positive ruminators may perceive the negative feelings brought about by depression as a signal for self-growth and change (82). By reflecting on their own thinking patterns, behavioral habits, and life experiences, they attempt to tap into potential creative resources. For example, they may gain more opportunities for introspection from the loneliness induced by depression, thereby stimulating an in-depth exploration of the inner world and providing unique materials and perspectives for creative thinking. This positive cognitive restructuring process helps break the shackles of depressive emotions on creativity, enabling individuals to transcend the interference of negative emotions and unleash their creative potential to some extent. This suggests that when intervening in the issue of decreased creativity caused by mobile phone addiction, we should not only focus on alleviating depressive emotions but also pay attention to cultivating individuals’ positive rumination ability and guiding them to adopt positive cognitive strategies to deal with stress and setbacks in life.

### 4.4 Limitations and future directions

Although this study on the relationships between smartphone addiction, depression, rumination, and creativity represents progress, it also has some limitations.

While this study identifies significant associations between variables, the cross-sectional design precludes conclusions about causal directions. Longitudinal research is needed to explore temporal dynamics, such as whether depression precedes smartphone addiction or vice versa, and how positive rumination may influence these trajectories over time. The questionnaire survey, although useful for large samples, suffers from self-report bias. Participants might have inaccurately reported mobile phone addiction and depression due to social desirability concerns. Future research could integrate behavioral experiments and physiological measures, such as electroencephalogram monitoring. By directly observing brain activity during phone use, we can better understand the neural mechanisms and their impact on creativity, thereby minimizing self-report errors. While efforts were made to diversify the sample, the use of convenience sampling may constrain the generalizability of the results. Subsequent studies will broaden the sampling framework to address this limitation. We examined the mediating and moderating roles of depression and rumination, other variables may need to be considered. Social support networks and personality traits (e.g., openness and neuroticism) may also be involved.

Future research can adopt the triangulation method, integrating multiple sources of data to enhance the quality of the study. Behavioral observation data can be incorporated by objectively recording usage behaviors through mobile phone usage monitoring software, which can be cross - verified with self - report data. Physiological measurement data can also be introduced, using fMRI and EEG technologies to monitor brain activities and provide evidence of neural mechanisms. Meanwhile, it is recommended to conduct longitudinal studies to track the dynamic changes of variables and clarify the causal sequence, and carry out experimental studies to test causal relationships through group - based interventions. The coordinated use of multiple methods will deepen the exploration of relationships among smartphone addiction, positive rumination, and creativity, and strengthen theoretical and practical foundations.

When applying the research findings, it is necessary to consider the characteristics of Chinese culture. In a collectivist cultural context, individual creativity often serves collective goals. The support from family and social networks is of great significance in resisting smartphone addiction and maintaining mental health and creativity. Therefore, when applying the research results in fields such as education and psychological intervention, elements of collective cooperation and family support should be integrated to enhance the adaptability and effectiveness of interventions. Cross - cultural comparative research can be carried out. By comparing China with Western individualistic cultures or other cultural regions, we can explore how cultural factors moderate the relationships among smartphone addiction, positive rumination, and creativity, and build a universal theoretical model. Meanwhile, exploring effective intervention measures in different cultural backgrounds can promote theoretical development and practical applications on a global scale.

## Data Availability

The raw data supporting the conclusions of this article will be made available by the authors, without undue reservation.
